# Cost impact of a non-invasive, portable device for patient self-administration of chronic migraine in a UK National Health Service setting

**DOI:** 10.1186/s40064-016-2924-8

**Published:** 2016-08-03

**Authors:** B. Brüggenjürgen, T. Baker, R. Bhogal, F. Ahmed

**Affiliations:** 1 Institute for Health Economics, Steinbeis-University, Berlin, Germany; 2 Institute for Social Medicine, Epidemiology and Health Economics, Charité University Medical Center, Berlin, Germany; 3 eNeura Inc., Sunnyvale, CA USA; 4 Neurosciences Business Unit, Hull and Yorkshire Hospitals NHS Trust, Hull, UK; 5 Hull York Medical School, Hull, UK

**Keywords:** Chronic migraine, Botox, TMS, Risk sharing, Economic analysis, Incremental costs, Pay for performance, Self-administration

## Abstract

**Background:**

Chronic migraine (CM) is a neurological disorder associated with substantial disability. Botulinum toxin type A (Botox) is an approved and effective preventive treatment option for adult patients with CM. Transcranial magnetic stimulation (TMS) is an alternative treatment device delivering a brief pre-set magnetic pulse used for self-administration by the patient at home. Despite being available in a risk share scheme TMS is perceived to be more costly in the UK. The objective of this study was to analyse the incremental costs of TMS compared to Botox in refractory CM patients both for a UK individual funding request setting as well as for an average UK specialist center setting.

**Methods:**

Cost impact results were derived from a decision-tree model simulating treatment pathways over 1 year. Costs were applied from the most recently available UK data sources. Sensitivity analysis was performed for all variables.

**Results:**

Based on published utilisation data 45.5 % of CM patients would continuously receive Botox over 1 year, whereas 53.7 % of TMS patients would be still on treatment at the end of year one. Total costs of Botox treatment accrue to £2923 in an individual funding request NHS cost setting, whereas TMS treatment results in £1466 in the first year. Applying a time-based NHS cost setting expenditures accrue to £1747 for the Botox treatment and to £1361 for the TMS treatment. In both cost settings variation of cost assumptions did have a minor impact on the cost increment from Botox to TMS.

**Conclusion:**

The current risk share based remuneration model of TMS allows the UK NHS to reimburse only the cost of those patients experiencing reduction in migraine days resulting in lower costs for treating migraine attacks. Treatment of chronic refractory migraine using TMS implies a substantial cost reduction potential for the management of chronic treatment of refractory migraine patients compared to conventional Botox treatment.

## Background

Chronic migraine (CM) is a neurological disorder associated with substantial disability (Blumenfeld et al. 2011; Bigal et al. 2008; Harwood et al. 2004; Munakata et al. 2009; Natoli et al. 2010). CM is defined as experiencing headaches on at least 15 days per month for ≥3 months, where ≥8 of those days per month are with migraine (Headache Classification Committee of the International Headache 2013). Refractory CM is a long-term disease, affecting around 610,000 people alone in the UK (Natoli et al. 2010; Ahmed 2011). Patients with CM are impacted by lower health-related quality of life and are more likely to suffer from severe disability (Blumenfeld et al. 2011; Harwood et al. 2004; Munakata et al. 2009; Lipton and Bigal 2003). This resulted in a low number of migraine prophylaxis trials as CM patients being considered to be too highly disabled and treatment resistant (Dodick et al. 2010).

Costs are related both to acute drug treatment as well as to prophylaxis and discontinuation is a relevant factor for the latter. CM patients consume more direct healthcare resources than those with episodic migraine, such as acute medication use, physician visits and hospitalisations, along with a significant reduction in workplace productivity (Blumenfeld et al. 2011; Harwood et al. 2004; Munakata et al. 2009; Lipton and Chu 2009). Only few treatment options are available for refractory chronic migraine patients.

Botulinum toxin type A (Botox) is an approved and effective preventive treatment option for adult patients with chronic migraine (Aurora et al. 2011). Botox injections are administered intramuscularly by a trained physician to between 31 and 39 sites divided across 7 specific head and neck muscle areas every 12 weeks in a specialist outpatients hospital clinic setting. According to NICE guidelines eligible patients for Botox receive at least two consecutive cycles for treatment irrespective of whether they are responsive to treatment in the first cycle (NICE 2012).

Non-invasive neuromodulation devices are alternative treatment options in acute and preventive migraine therapy (Barbanti et al. 2015; Barker et al. 1985; Bhola et al. 2015). Spring TMS™ uses portable, single pulse transcranial magnetic stimulation technology. It is an approved and effective patient use device for both acute and preventive treatment of migraine. TMS technology was invented in the UK by the Royal Hallamshire NHS Hospital and University of Sheffield demonstrating the first stimulator in 1985 (Barker et al. 1985). In results from a UK Post Market Pilot programme Spring TMS demonstrated safety, efficacy and very good tolerability in acute migraine for daily and preventative treatment use (Bhola et al. 2015). The Spring TMS™ device is placed against the back of the head for less than a second to deliver a very brief pre-set magnetic pulse. The magnetic field passes through the skull and tissue non-invasively and without discomfort to induce electrical currents along the cortex of the brain. Spring TMS™ is designed to be used for self-administration by the patient at home and requires minimal patient training.

The National Institute for Clinical Excellence (NICE) has approved TMS for clinical use through its Interventional Procedure Guidance (IPG) 477 January 2014. However, NICE has not yet completed a technology (cost) appraisal or issued a Technology Appraisal Guidance (TAG). TMS is available under a risk sharing programme in the UK, where the initial quarterly usage evaluates patient response to treatment. This first quarter is financed by the supplier. The subsequent quarter usage is to be paid by the NHS only for those patients benefiting from TMS observed as a reduction in migraine day frequency and or severity.

TMS addresses current NHS strategy to deliver services close to the patient and out of the hospital. The use of TMS supports Kings Fund first priority of self-management for commissioners to deliver a more efficient and effective system with improved outcomes for the patient including pain management.

However, the general perception about TMS among healthcare professionals and funding authorities is that the treatment is more expensive than existing therapies such as Botox and as a consequence many primary care physicians and migraine clinics have been reluctant to prescribe the device and have taken an Individual Funding Request route for TMS in exceptional cases. Hence, the objective of this cost analysis was to evaluate the incremental cost impact of TMS by comparing two real life treatment costs scenarios of Botox and TMS over 1 year both from an individual funding request perspective as well as from an average time-derived bottom-up UK cost center setting.

## Methods

Our analysis outlines direct costs that accrue over time in patients with chronic refractory headache using either the pharmacologic injections or the risk sharing device-based preventive TMS treatment. The present study employs a cost analytic approach (Drummond et al. 2005). A decision analytic model was developed to outline the incremental cost impact of the risk-sharing approach of TMS compared to the traditional costing approach of Botox based on two alternative UK NHS costing settings. Modeling was performed using Treeage 2015.

### Model design

**Fig. 1:**
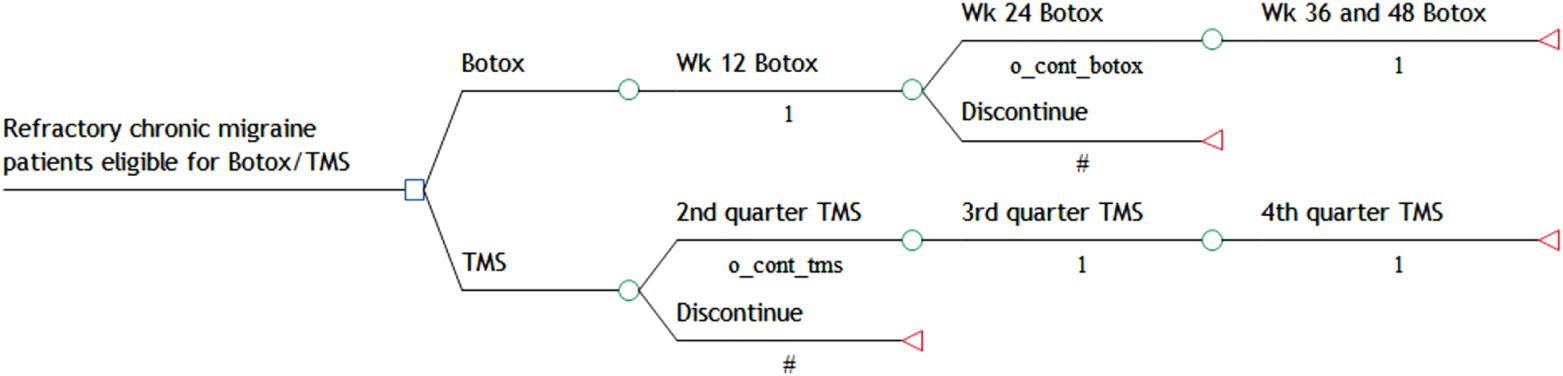
Decision tree model for two alternative prophylactic treatment approaches in refractory chronic migraine patients in the UK

The model assumes that after being considered eligible patients are treated consecutively over 5 cycles of Botox or four quarter of TMS unless patients discontinue treatment (Fig. 1). Botox patients are assumed to be treated at least over 2 consecutive 12 week-cycles according to the currently applied treatment scheme in the UK. TMS patients are subject for discontinuation after the initial quarter. It is assumed that those patients continuing either on Botox or TMS will remain on treatment until the end of the year.

### Outcome and treatment attrition

For both treatment alternatives a reduction in resource utilisation when experiencing headache days for those patients responding of 49 % for Botox and 47 % for TMS were assumed (Bhola et al. 2015; Rothrock 2011). Responder rates were assumed to be identical with continuation rates. Values were based on published UK trial data resulting in 45.5 % for Botox (Khalil 2015a) and 53.7 % for TMS (Bhola et al. 2015).

### Costs

In order to reflect potential NHS cost perspectives a bottom-up costing both for a scenario based on an individual funding authorisation perspective as well as an average time-based NHS resource use were calculated: Specialist center published fees of £650 per cycle for administering treatment, drug costs and follow-up visits and £140 for initial neurologist consultation were applied for the individual funding authorisation (Hull Royal Infirmary K-U-H 2012). For the time-derived cost setting the values were based on expert advice and NICE Botox appraisal values (NICE 2011). Hence, Botox resource consumption in the initial cycle would require 30 min specialists time resulting in £70.00 with unit cost of £2.33 applied (NICE 2011; Curtis 2014). Administering TMS would require one follow-up 15 min visit in the first quarter resulting in £35.00 (Table 1). Treatment period prorated administration and drug or device costs for the CM preventive treatment with either Botox or TMS were calculated for a maximum of 5 cycles and four consecutive quarters, resp. 12-weeks Botox drug costs of £276.40 (NICE 2011) and quarterly TMS costs of £450 (eNeura, Manufacturer) were applied.

**Table 1:** Costs and resource use assumptions

Variable	Individual scenario	Time-derived	Definition	Source
Administration costs Botox initial cycle	£513.60	£70.00	Spire: Treatment (£650 incl. follow-up visits and drug) + initial adminstration (£140) − drug costs/Time-based: Consultant visit lasts 30 min plus 15 min nurse time	http://www.spirehealthcare.com/hull/news/botox-for-migraine-treatment/. Accessed 14/02/2016, PSSRU, unit costs of health and social care 2014 (Curtis 2014)
Administration costs Botox every 12 weeks	£373.60	£60.00	Spire Hull: Treatment (£650, incl. follow-up visits and drug) - drug costs/Time-based: Consultant visit lasts 15 min plus 15 min nurse time	http://www.spirehealthcare.com/hull/news/botox-for-migraine-treatment/. Accessed 14/02/2016, PSSRU, unit costs of health and social care 2014 (Curtis 2014),
Botox drug costs per 12 weeks cycle	£276.40	£276.40	Drug costs	NICE Botox appraisal
Botox drug costs for 48 week cycle	£276.40	£276.40	Drug costs	NICE Botox appraisal
Administration costs TMS in initial quarter	£140.00	£35.00	Spire Hull: Initial consultation fee £140/Time-based: Consultant visit lasts 15 min	http://www.spirehealthcare.com/hull/news/botox-for-migraine-treatment/. Accessed 14/02/2016; PSSRU (Curtis 2014), Personal communication Hull
Administration costs TMS in follow-up quarter	0	0	Device used at home by patient	
Costs TMS in initial quarter responder	0	0	Not applied due to risk share approach	Manufacturer
Costs TMS in follow-up quarter responder	£450.00	£450.00	Current sTMS costs	Manufacturer
Costs 1 GP Visit	£45.63	£45.63	Surgery consultation last 11.7 min £3.90 per min (with qualification costs) incl. direct staff (Table 10.8b)	PSSRU, unit costs of health and social care 2014 (Curtis 2014)
Costs hospital inpatient stay migraine	£514.00	£514.00	NHS 2013/14 National Tariff—Patients continuing on Botox and TMS were assumed to be in need for furhter neurologists visits; PA04B applied for dicontinuing	https://www.gov.uk/government/collections/payment-by-results-2013-14. Accessed 14/02/2016
Costs neurologist visit	£128.00	£128.00	National tariff information spreadsheet-index: Neurology and Neurosurgery outpatient attendances	https://www.gov.uk/government/collections/payment-by-results-2013-14. Accessed 14/02/2016
Costs emergency department visit	£78.00	£78.00	National Tariff—VB09Z	https://www.gov.uk/government/collections/payment-by-results-2013-14. Accessed 14/02/2016
Costs Triptan use per attack	£1.08	£1.08	Weighted average for the mean cost of 1 triptan tablet in NHS England 2014	http://www.hscic.gov.uk/catalogue/PUB17274/pres-cost-anal-eng-2014-rep.pdf and Botox appraisal 2011
Number of A&E events per quarter	0.51	0.51	UK specific data from IBMS, Table 3	Bloudek et al. (2012)
Number of GP visits for migraine per quarter	1.44	1.44	UK specific data from IBMS, Table 3	Bloudek et al. (2012) and Blumenfeld et al. (2011)
Number of hospital inpatient stays per quarter	0.09	0.09	UK specific data from IBMS, Table 3	Bloudek et al. (2012)
Number of neurologists visit per quarter	0.43	0.43	UK specific data from IBMS, Table 3	Bloudek et al. (2012)
Number of Triptans used per quarter	7.29	7.29	7.29 triptans used for patients more than 15 attacks	NICE Botox Appraisal (2011): Table 6.17 page 160
Probability Botox continuation	45.5 %	45.5 %	Responder after cycle 2 based on Khalil et al. (2015b)	Khalil et al. (2015b)
Probability TMS continuation	53.7 %	53.7 %	Discontinuer minus those discontinuing due to lack of funding	Bhola et al. (2015)
Botox resource use reduction per responder 12 weeks	49.7 %	49.7 %	Observed reduction rate in observational trial	Rothrock (2011), Table 3
TMS resource use reduction per responder quarter	46.7 %	46.7 %	Observed reduction rate in observational trial	Bhola et al. (2015), Table 3

Treatment cost components were covering costs for physician visits due to migraine, cost of hospital visits due to migraine, cost of emergency room visits due to migraine and symptomatic treatment costs per day with headache. UK specific resource use consumptions for standard treatment of CM patients were obtained from the International Burden of Migraine Study (IBMS) (Bloudek et al. 2012). Resource unit valuation was based on 2013 NHS tariff information if not stated differently either from the 2013–2014 tariff information spreadsheet or based on the PSSRU (Curtis 2014; Department_of_Health 2013) (Table 1).

### Sensitivity analyses

Deterministic one-way sensitivity analyses were performed both for the individual funding request setting as well as for the time-derived NHS setting. Apart from one variable (where Botox drug costs in week 48 for the low value assumes a 50 % share for the first 6 weeks only) all other parameter were varied at 10 %. Sensitivity analyses have been reported in an incremental tornado diagram highlighting the impact of the assumptions on the potential incremental cost difference of the two preventive treatment choices in both settings.

## Results

Based on the outlined assumptions 45.5 % of CM patients would continuously receive Botox over 1 year. 53.7 % of TMS patients would be still on treatment at the end of year one and are responding. Total costs of the Botox treatment accrue to £2923, whereas TMS treatment results in £1466 in the first year. Applying the time-based cost setting expenditures accrue to £1747 for the Botox treatment and to £1361 for the TMS treatment.

### Sensitivity analyses

**Fig. 2:**
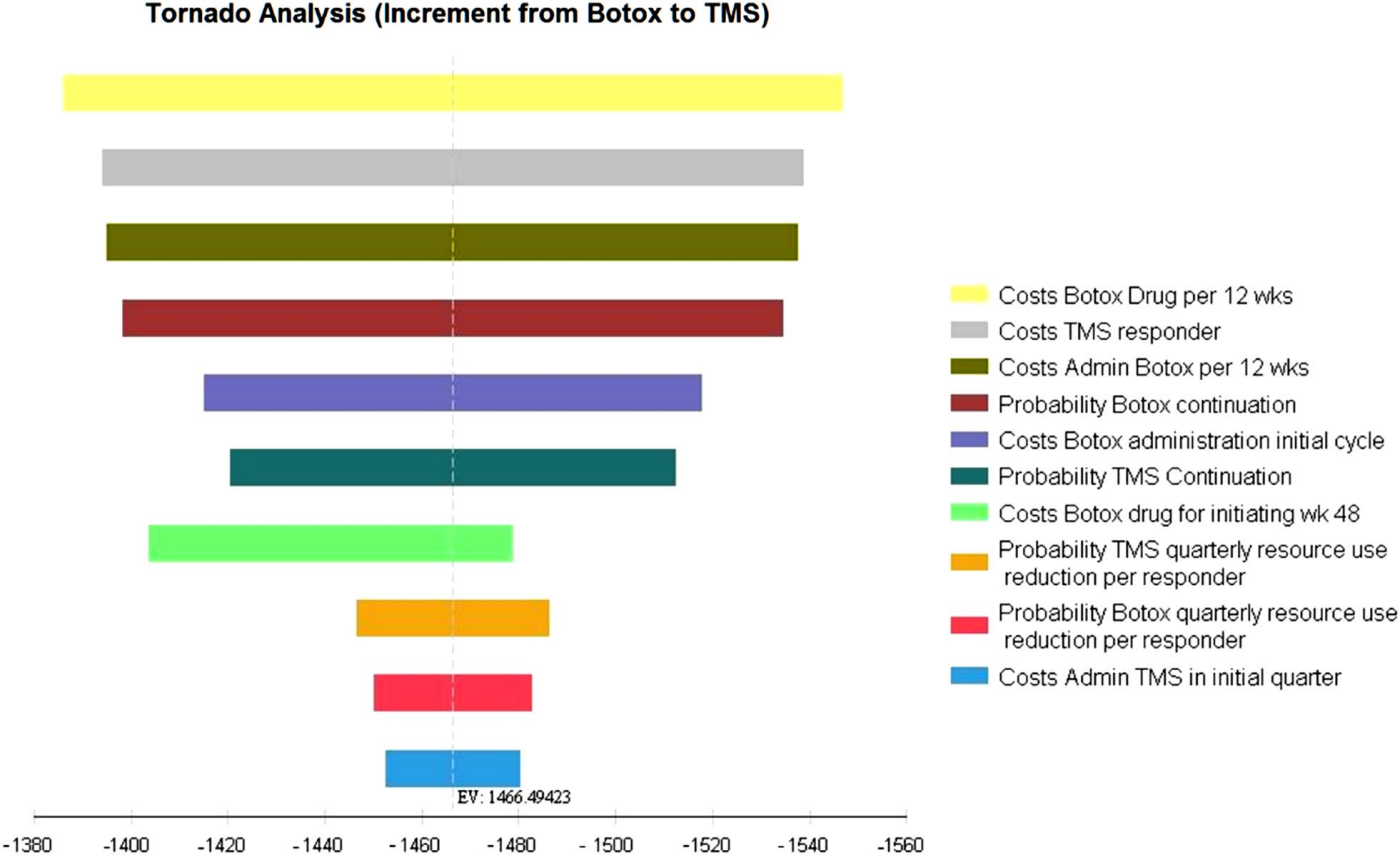
Incremental sensitivity analysis Botox versus TMS for individual funding request setting (increment from Botox to TMS in £; *negative values* indicate savings)

Sensitivity analyses are reported from an incremental cost perspective. The base case increment from Botox to TMS is −£1466 for the individual funding request setting and −£386 for the time-based setting. When varying values at 10 % in the individual funding request setting Botox drug costs were the most influential variable (Range −£1386 to −£1547) followed by the cost for treating TMS responder (range −£1394 to −£1539) (Fig. 2).

**Fig. 3:**
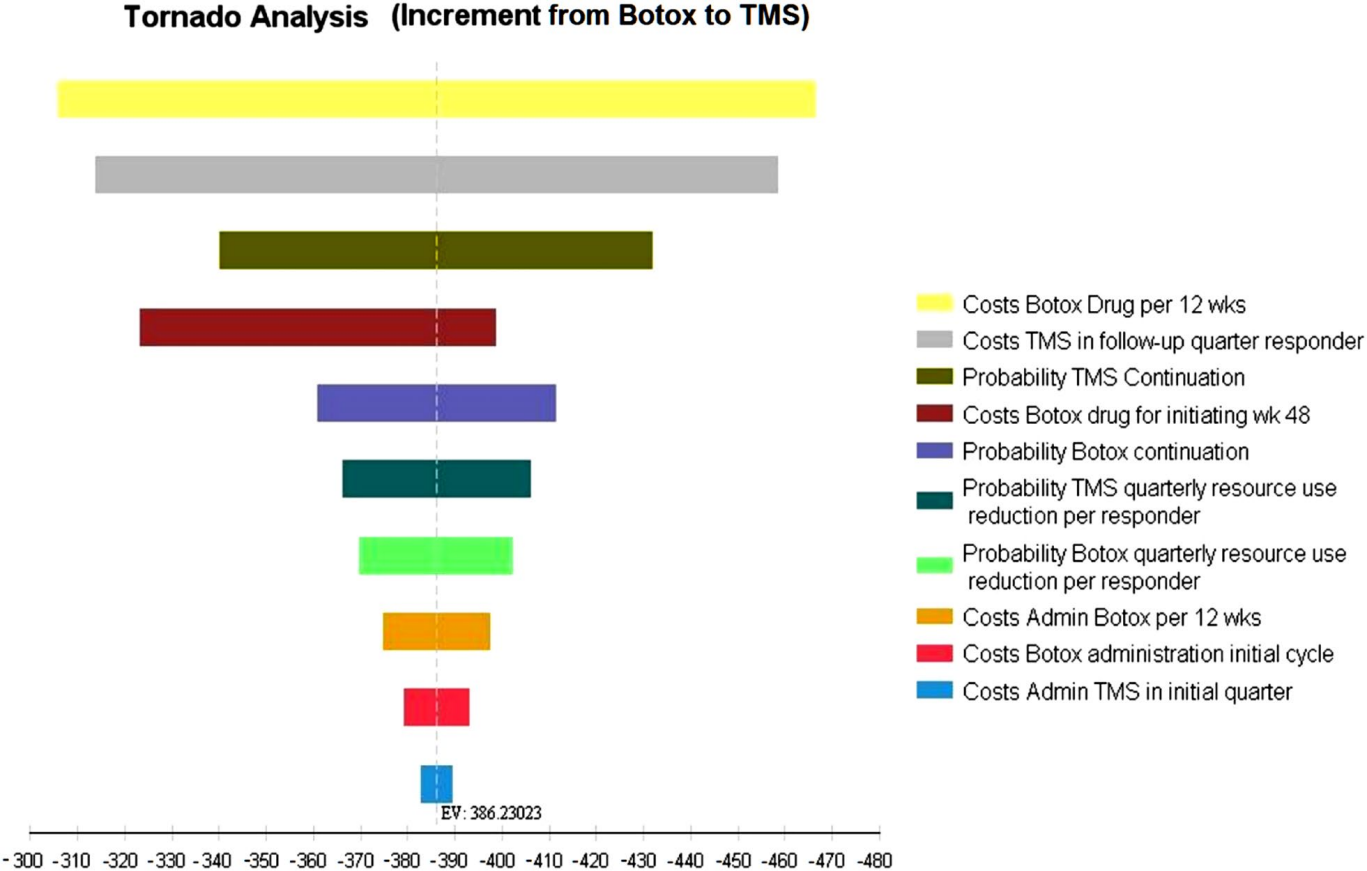
Incremental sensitivity analysis Botox versus TMS for time-based setting (increment from Botox to TMS in £; *negative values* indicate savings)

The incremental changes due to variation of values in the time-based setting are most influenced when varying the drug costs of Botox (range −£467 to −£306) followed by the cost for TMS per quarter (range −£459 to −£314) (Fig. 3).

## Discussion

This cost impact analysis compares 12-weekly Botox injections versus on demand patient applied TMS applications in refractory chronic migraine patients. Total annual cost of a chronic migraine patient in the UK accrue to £3718 and would result into a total UK cost burden of £2.2 billion when assuming a prevalence of 0.91 % (Bloudek et al. 2012; Buse et al. 2012). From a NHS UK perspective, a potential for cost reduction of £1466 per patient in an individual funding request setting and £386 in a time-based average NHS cost setting was observed.

Our cost calculations are in the range of other cost analyses in chronic refractory patients conducted in other countries. The Scottish Medicines Consortium reports an incremental cost of £1394 when treating CM patients in addition with Botox (Scottish Medicines Consortium Submission 2011). A US analysis calculated annual cost related to Botox treatment of 4902 US $, which is higher compared to both the individual funding request setting as well as the time-based average cost setting (Rothrock 2011). A German economic analysis of chronic cluster headache treatment assessed a comparable cost decrement of €414 for a non-invasive neuromodulatory technique in addition to standard of care (Morris et al. 2016).^.^

Resource consumption data related to the treatment of chronic migraine patients in real life are sparse. Estimates of medical resource use were drawn from the International Burden of Migraine Study (IBMS) (Blumenfeld et al. 2011). Impact of assumptions on cost differences was tested with varying the underlying assumptions for all variables at 10 %, which resulted in only small deviations from the base case highlighting savings for TMS. Even when pro-rating the 48-week Botox drug costs, resulting in a bisection of the Botox drug cost in the fifth cycle, the increment from TMS to Botox remains negative at £1404. Hence, the size of the incremental differences is very robust to realistic changes of all parameters.

TMS is available under a pay for performance scheme in the UK, where the initial test quarter is free and the following quarters only have to be paid for responders. Risk sharing approaches in healthcare are not appropriate in all settings. Schemes should have realistic time scales and low administrative burden and should be based on sound evidence (Adamski et al. 2010). The TMS scheme is straightforward and places no further administrative burden on decision makers and hence is suitable for achieving cost reduction potentials in healthcare. However, there is presumably a low awareness of the risk sharing approach related to TMS, as still high cost perceptions anticipated for the non-drug treatment approach from a decision-maker point of view exist. Our analysis shows major cost reduction potentials for TMS user.

Our modelling might comprise some limitations: The current model does no account for potential differences in side effects, which are more pertinent with Botox being associated in a UK real-life setting with neck stiffness (16 %), injection site pain (15 %) and ptosis (9 %) (Khalil et al. 2015b). TMS patients reported minor neurologic symptoms only lasting up to 30 min after applying the first pulse (20 %) (Bhola et al. 2015). As with other cost analyses in CM patients our decision tree model only covers the initial year and hence those receiving a longer treatment period might not adequately be acknowledged.

A strength of our model is the focus on real life settings. Hence, with an individual specialist centre cost scenario and the most recent UK real life data selected our model in particular addresses the decision-maker’s information needs of choosing efficient treatment options for severely impacted chronic migraine patients. Our cost assumptions are conservative as the expertise for the evaluation of eligible chronic migraine patients and for the individual treatment management decisions still remains scare in the UK and hence might result even in higher resource utilisation in those centres not being experienced with severe chronic migraine patients. In the Hull specialist centre the management of those 254 patients treated with Botox from 2010 to 2013 (Khalil et al. 2015b) would have resulted in a cost reduction of £372,364 (individual funding request assumptions) or £98,044 (time-based average NHS assumptions) if having been treated initially with TMS.

## Conclusion

Both Botox and transcranial magnetic stimulation provide effective treatment approaches in patients with treatment-refractory chronic migraine in UK settings (Bhola et al. 2015; Khalil et al. 2014). TMS is perceived to induce higher costs compared to the pharmaceutical approach. Our decision analytic model demonstrates that treatment of chronic refractory migraine using TMS implies a substantial cost reduction potential for the management of treatment of chronic refractory migraine patients compared to conventional BOTOX treatment. The current risk share based remuneration model of TMS allows the National Health Service (NHS) to bear only the cost of those showing reduction in migraine days resulting in lower costs. Risk sharing is a relevant and future-oriented approach to performance based management in healthcare systems. Particularly in unclear efficacy situations risk sharing is providing an efficient approach for paymasters in healthcare and thus manages to balance patient needs and budgets when introducing innovative treatments.
